# Advancements in Artificial Intelligence for Dentomaxillofacial Radiology—2nd Edition: From Proof of Concept to Generalizable and Clinically Trustworthy AI

**DOI:** 10.3390/diagnostics16213681

**Published:** 2026-11-07

**Authors:** Kaan Orhan, Andre Luiz Ferreira Costa, Sérgio Lúcio Pereira de Castro Lopes

**Affiliations:** 1 Department of Dentomaxillofacial Radiology, Faculty of Dentistry, Ankara University, Ankara 06560, Turkey; 2 Department of Oral Diagnostics, Faculty of Dentistry, Semmelweis University, 1088 Budapest, Hungary; 3 Medical Design Application and Research Center (MEDITAM), Ankara University, Ankara 06000, Turkey; 4 Postgraduate Program in Dentistry, Cruzeiro do Sul University (UNICSUL), São Paulo 08060-070, SP, Brazil; alfcosta@gmail.com; 5 Department of Diagnosis and Surgery, São José dos Campos School of Dentistry, São Paulo State University (UNESP), São José dos Campos 12245-000, SP, Brazil; sergio.lopes@unesp.br

Only a few years ago, the central question in artificial intelligence (AI) for dentomaxillofacial radiology was whether algorithms could match human experts at narrowly defined diagnostic tasks [1,2,3]. The thirteen contributions gathered in this second edition of the Special Issue “Advancements in Artificial Intelligence for Dentomaxillofacial Radiology” suggest that the field is now confronting a harder set of questions: whether accuracy survives across devices, institutions, and patient populations; whether AI assistance measurably changes what clinicians do; and whether new classes of models, such as radiomics–deep learning hybrids, lightweight edge networks, and large language models (LLMs), can be trusted at the chairside [4]. This closing editorial shows how the contributions to this edition address these questions and sketches the research agenda that follows from them.

A defining characteristic of this edition is its focus on clinically deployed or commercially available systems rather than laboratory prototypes alone. One contribution evaluated the AI-based Diagnocat system for periapical lesion detection on panoramic radiographs, reporting perfect specificity (1.00) alongside moderate sensitivity (0.78), with performance varying markedly by tooth type—a salient reminder that aggregate metrics can conceal clinically important regional weaknesses [5]. A similar profile has been reported outside this Special Issue: in an external validation of two FDA-cleared decision support systems for caries and periodontal bone loss, both showed high specificity and negative predictive value, supporting their role as adjunctive screening aids rather than stand-alone diagnostic tools [6]. AlGhaihab et al. went a step further in the periodontal domain by asking the consequential question of whether AI assistance actually changes clinician performance. In their reader study with ten general dentists, deep learning support yielded no statistically significant improvement in diagnostic accuracy, with area-under-the-curve differences of only 0.031 for periapical and −0.009 for bitewing radiographs [7]. By contrast, a randomized controlled trial of AI-assisted detection of periapical radiolucencies found a modest gain in accuracy, driven mainly by fewer false-positive findings, with the largest benefit among less experienced dentists [8]. In orthodontics, another contribution to this Special Issue evaluated the clinical utility and time efficiency of AI-supported and app-aided cephalometric analysis platforms in comparison with conventional manual tracing. Together, these studies reframe AI tools as adjuncts whose value must be demonstrated at the level of the clinician–machine team, not at the level of the algorithm alone.

If the first edition of this Special Issue showcased algorithmic capability, this second edition interrogates its portability. Özcan and Allaf trained four deep learning architecture families on two independently sourced cephalometric datasets and showed that in-domain rankings did not predict cross-domain rankings: the most accurate in-domain model suffered among the largest performance drops, cross-dataset errors rose by 155–426%, and clinically decisive landmarks (A-point, B-point, Nasion, and Menton) exceeded the 2 mm acceptability threshold out-of-domain, whereas balanced multi-source training recovered most of the loss [9]. Another contribution provides a complementary, encouraging example: a U2-Net architecture for detecting separated endodontic instruments, developed on panoramic radiographs pooled from multiple imaging systems, achieved robust segmentation performance (Dice coefficient 0.849), although device-stratified external testing has yet to be performed. Two recent external validation studies of commercial CBCT tooth segmentation software point in the same direction: accuracy was largely maintained across five CBCT systems, with little influence of the scanner itself, whereas patient-related factors such as orthodontic brackets, endodontic treatment, and high-density artifacts increased the need for manual refinement [9,10]. At the evidence-synthesis level, Hashim et al. appraised sixteen publications on deep learning for alveolar bone loss on intraoral radiographs and delivered a sobering verdict: only two studies performed external validation, none was prospective, and the certainty of evidence was rated very low [11].

The methodological and clinical breadth of the field also continues to expand. Çetin et al. fused handcrafted radiomic features with a purpose-built convolutional architecture (RadC-CNN) to differentiate radicular cysts from periapical granulomas on CBCT—entities that are notoriously difficult to separate preoperatively—achieving 90% accuracy against histopathologically confirmed diagnoses [12]. Across jawbone lesions more broadly, a recent meta-analysis reported consistently high specificity but widely varying sensitivity, and noted that limited external validation and transparent reporting still restrict clinical interpretability [13]. Another contribution tested whether general-purpose LLM-based chatbots (GPT-4o, GPT-o4-mini-high, and GPT-o1-pro) could estimate bone age and growth stage directly from hand–wrist radiographs without domain-specific training; the predictions correlated significantly with expert reference values, although agreement remained modest, positioning such models as accessible adjuncts rather than replacements. Exploring the frontier of generative and language-driven artificial intelligence, emerging investigations in dentistry and radiology have begun evaluating general-purpose foundation models and multimodal vision–language architectures for clinical triage, automated interpretation, and decision support [14,15]. However, as emphasized in recent radiological best practices, clinical use of such generative models requires rigorous safeguards against confabulation, limited reproducibility, stochastic drift, and data privacy risks [16]. Addressing daily diagnostic, prosthetic, and surgical challenges, further contributions to this Special Issue expanded automated analysis across modalities—demonstrating deep learning pipelines for dental implant system identification, automated segmentation of delicate anatomical structures such as the infraorbital canal, AI-assisted volumetric planning for maxillary sinus floor augmentation, and lightweight explainable frameworks for pediatric infraocclusion detection.

Future research should therefore prioritize the following directions:

• Prospective, multicenter, multi-device external validation, with local acceptance testing on the receiving institution’s own images before clinical reliance [9,11,17].
• Clinically anchored reference standards—clinical attachment levels, surgical confirmation, or histopathology—to replace annotation-only ground truth [6,11].
• Human-in-the-loop reader studies that measure the performance of clinician-plus-AI teams, explicitly addressing automation bias, workflow effects, and patient outcomes [7,8,18].
• Open, multi-device benchmark datasets with device-stratified reporting, enabling fair comparison beyond single-dataset leaderboards [9,19,20].
• Responsible exploration of foundation and vision–language models, with explicit calibration, hallucination control, and privacy-preserving training strategies such as federated learning [16].
• Full adoption of AI-specific reporting and risk-of-bias standards (CLAIM, TRIPOD-AI, CONSORT-AI, and PROBAST-AI) and early engagement with regulatory approval pathways [21,22,23,24,25,26,27].

Like its predecessor, this Special Issue was enriched by contributions from multiple continents and disciplines, spanning periodontology, endodontics, orthodontics, implant dentistry, oral and maxillofacial surgery, and pediatric dentistry. We sincerely thank all authors, reviewers, and the editorial team of *Diagnostics* for their commitment. Taken together, these thirteen papers show that the question is no longer whether AI can interpret dentomaxillofacial images, but how to ensure that it does so reliably, transparently, and equitably in everyday clinical practice. We hope this collection accelerates the transition from proof of concept to validated, trustworthy clinical integration.
